# The Effect of Roux-en-Y vs. Omega-Loop Gastric Bypass on Liver, Metabolic Parameters, and Weight Loss

**DOI:** 10.1007/s11695-016-2083-6

**Published:** 2016-03-22

**Authors:** Renate Kruschitz, Maria Luger, Christian Kienbacher, Michael Trauner, Carmen Klammer, Karin Schindler, Felix B. Langer, Gerhard Prager, Michael Krebs, Bernhard Ludvik

**Affiliations:** 1 Division of Endocrinology and Metabolism, Department of Internal Medicine III, Medical University of Vienna, Vienna, Austria; 2 Special Institute for Preventive Cardiology And Nutrition – SIPCAN save your life, Salzburg, Austria; 3 Division of Gastroenterology and Hepatology, Department of Internal Medicine III, Medical University of Vienna, Vienna, Austria; 4 Division of General Surgery, Department of Surgery, Medical University of Vienna, Vienna, Austria; 5 Department of Internal Medicine I with Diabetology, Endocrinology and Nephrology Rudolfstiftung Hospital, Karl Landsteiner Institute for Obesity and Metabolic Diseases, Vienna, Austria

**Keywords:** Gastric bypass, Morbid obesity, Liver parameters, Metabolic parameters, Weight loss

## Abstract

**Background:**

Omega-loop gastric bypass (OLGB) results in weight loss (WL) but data on its impact on liver and glucose metabolism compared to Roux-en-Y gastric bypass (RYGB) is lacking. Therefore, the aim of this study was to compare the development of hepatic and metabolic markers as well as WL between the above-mentioned surgical groups during the first postoperative year.

**Methods:**

We retrospectively evaluated the respective parameters in non-diabetic morbidly obese patients who underwent either RYGB (*n* = 25) or OLGB (*n* = 25).

**Results:**

Compared to RYGB, OLGB showed a greater WL percentage. Liver transaminases dropped in RYGB, while rose in OLGB. No correlation between aspartate transaminase, alanine transaminase, and WL could be detected. Gamma-glutamyltransferase decreased significantly in RYGB over the first 3 months, while it increased in OLGB. We found higher levels of triglycerides, insulin, homeostasis model assessment of insulin resistance (HOMA2-IR), and liver fat percentage in RYGB at baseline, despite matching the groups for age, sex, and BMI. Those differences disappeared, except for triglycerides, within 1 year. All metabolic parameters correlated with WL.

**Conclusion:**

OLGB results in greater WL but transiently deteriorated several liver parameters in the first postoperative year. This was not associated with WL. The impact of these results on hepatic outcomes such as non-alcoholic steatohepatitis and fibrosis progression requires further studies. In both groups, improved insulin resistance and sensitivity were correlated with higher WL and lower liver fat percentage, respectively.

## Introduction/Purpose

Bariatric surgery is the most successful treatment of morbid obesity [1] since it is associated with effective long-term weight loss (WL) and decreases overall mortality [2]. At present, three categories of bariatric procedures are in use: (a) purely gastric restriction (e.g., gastric banding, sleeve gastrectomy); (b) gastric restriction with a mild malabsorptive effect, represented by Roux-en-Y gastric bypass and omega-loop gastric bypass; and (c) gastric restriction with an extensive malabsorptive effect such as the biliopancreatic diversion [3]. The extent of WL is in part related to the complexity of the bariatric procedure [4]. A small number of studies have compared the results of Roux-en-Y gastric bypass, which is still the gold standard for many bariatric teams [5], and omega-loop gastric bypass. Regarding previous data, it became evident that omega-loop gastric bypass is a bariatric procedure that demonstrates initial promising results in terms of WL, feasibility, and safety [6, 7]. However, little is known about the short- and long-term impact of this procedure, especially in terms of its impact on liver function and glucose metabolism.

Several studies investigating the effect of bariatric surgery on liver enzymes and non-alcoholic fatty liver disease (NAFLD) have shown an improvement of serum transaminases and hepatic histologic features after surgery [8–11]. From previous data, however, it became evident that rapid WL, as seen with bariatric surgery, can also adversely affect the liver [11]. Moreover, several case reports of patients with early hepatic failure after bariatric surgery are described in the literature [12–14].

The aim of this evaluation was to provide data on the development of hepatic and metabolic markers as well as on WL in morbidly obese patients undergoing either Roux-en-Y gastric bypass or omega-loop gastric bypass during the first 12 postoperative months to facilitate the design of studies in larger populations.

## Materials and Methods

### Preoperative Evaluation

For this investigation, we accomplished an analysis within the cohort study considering all consecutive bariatric patients in the outpatient clinic of the Division of Endocrinology and Metabolism in the General Hospital Vienna. The procedures used in this study were in accordance with the Declaration of Helsinki [15] and were approved and registered (no. 988/2011) by the Ethics Committee of the Medical University of Vienna. All participants provided their written informed consent prior to the study. A multidisciplinary team performed a combined workup to ensure that potential surgical candidates met the criteria for bariatric surgery [16]. The exclusion criteria were as follows: diabetes, earlier bariatric surgery, active malignancy during the past 5 years, myocardial infarction during the past 6 months, eating disorders, psychiatric problems contraindicating bariatric surgery, regular use of cortisone, alcohol (>20 g/day men, >10 g/day women) or drug abuse, and other severe illnesses. Daily alcohol consumption was evaluated during the preoperative dietary counseling on basis of the self-reported nutritional protocols.

### Patients

**Fig. 1:**
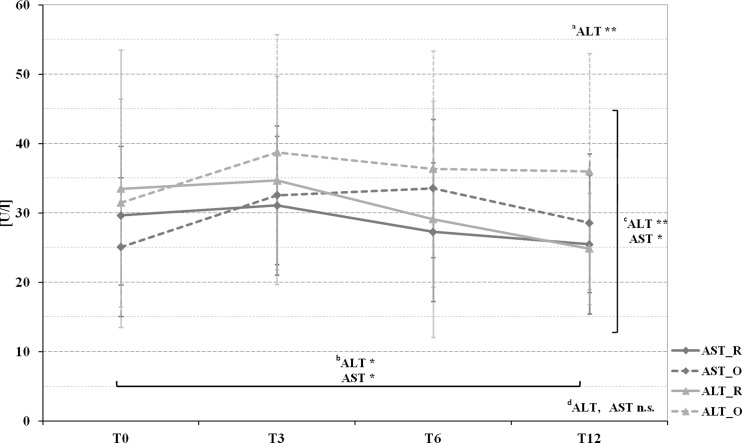
Pre- and postoperative mean protein and albumin levels in patients with Roux-en-Y gastric bypass (marked by a *full line*) and omega-loop gastric bypass (marked by a *dotted line*).^*a*^ *T* test comparing Roux-en-Y gastric bypass and omega-loop gastric bypass at baseline (T0), 3 (T3), 6 (T6), and 12 (T12) months. Linear mixed model (LMM), adjusted for baseline values, sex, and age for changes over ^*b*^time course, ^*c*^between groups, and ^*d*^changes over time course and between groups. *p < 0.05; **p < 0.01; ***p < 0.001; *n.s.* not significant

**Fig. 2:**
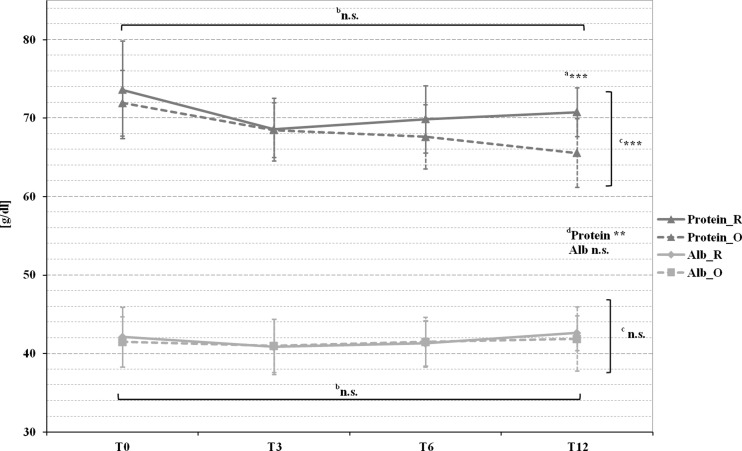
Pre- and postoperative mean AST and ALT levels in patients with Roux-en-Y gastric bypass (marked by a *full line*) and omega-loop gastric bypass (marked by a *dotted line*). ^*a*^ *T* test comparing Roux-en-Y gastric bypass and omega-loop gastric bypass at baseline (T0), 3 (T3), 6 (T6), and 12 (T12) months. Linear mixed model (LMM), adjusted for baseline values, sex, and age for changes over ^*b*^time course, ^*c*^between groups, and ^*d*^changes over time course and between groups. *p < 0.05; **p < 0.01; ***p < 0.001; *n.s.* not significant

**Fig. 3:**
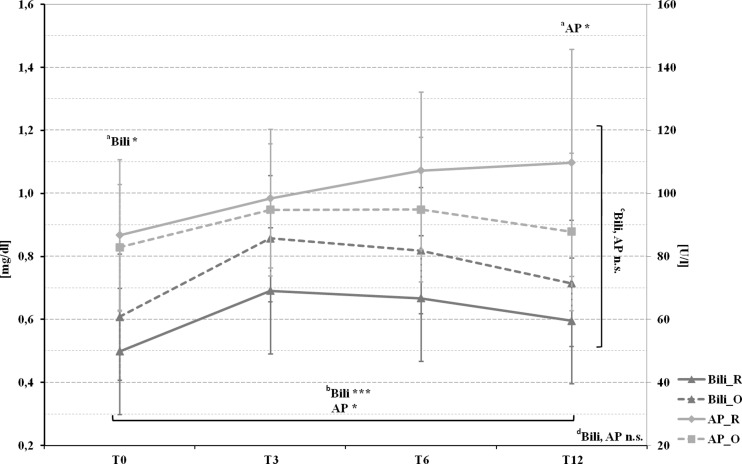
Pre- and postoperative mean bilirubin and alkaline phosphatase levels in patients with Roux-en-Y gastric bypass (marked by a *full line*) and omega-loop gastric bypass (marked by a *dotted line*). ^*a*^ *T* Test comparing Roux-en-Y gastric bypass and omega-loop gastric bypass at baseline (T0), 3 (T3), 6 (T6), and 12 (T12) months. Linear mixed model (LMM), adjusted for baseline values, sex, and age for changes over ^*b*^time course, ^*c*^between groups, and ^d^changes over time course and between groups. *p < 0.05; **p < 0.01; ***p < 0.001; *n.s.* not significant

**Fig. 4:**
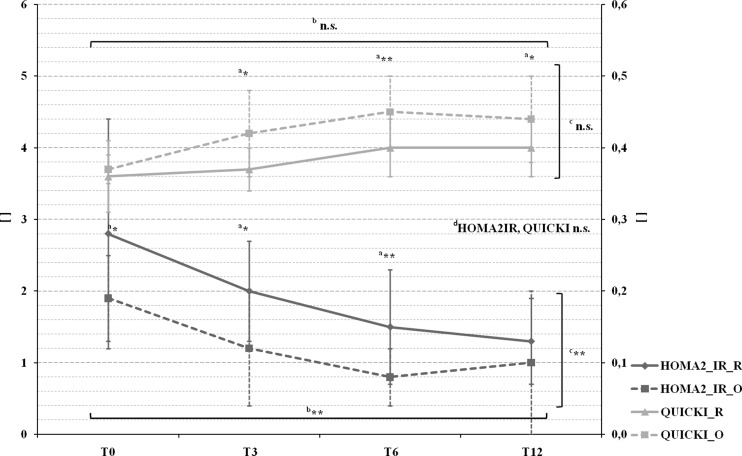
Pre- and postoperative mean HOMA2-IR and QUICKI levels in patients with Roux-en-Y gastric bypass (marked by a *full line*) and omega-loop gastric bypass (marked by a *dotted line*). ^*a*^ *T* test comparing Roux-en-Y gastric bypass and omega-loop gastric bypass at baseline (T0), 3 (T3), 6 (T6), and 12 (T12) months. Linear mixed model (LMM), adjusted for baseline values, sex, and age for changes over ^*b*^time course, ^*c*^between groups, and ^*d*^changes over time course and between groups. *p < 0.05; **p < 0.01; ***p < 0.001; *n.s.* not significant

From February 2011 to February 2013, a total of 86 patients underwent omega-loop gastric bypass [17]. Due to results on preliminary data that showed significant differences between diabetic and non-diabetic subjects [18], diabetic patients (*n* = 22) were not included in this analysis. In order to adequately compare the available data on omega-loop gastric bypass to the Roux-en-Y gastric bypass procedure, we matched patients by age, gender, and initial body mass index (BMI). Moreover, 36 patients with missing preoperative liver data and 3 with no adequate Roux-en-Y gastric bypass matches were excluded from the current analysis. Non-diabetic subjects who underwent either Roux-en-Y gastric bypass (*n* = 25, female = 92 %, 44.6 ± 10.3a, 125 ± 18 kg, BMI 45.6 ± 4.1) or omega-loop gastric bypass (*n* = 25, male = 88 %, 43.8 ± 13.1a, 128 ± 24 kg, BMI 45.3 ± 5.3) were analyzed. Baseline data are presented in Table 1 and Figs. 1, 2, 3, and 4.

**Table 1:** Evaluated liver, cholestatic variables, and weight loss of Roux-en-Y gastric bypass and omega-loop gastric bypass from T0 to T12

	T0					T3				T6				T12				*p* value**	*p* value**	*p* value**
		*n*	Mean	STD	p*	*n*	Mean	STD	p*	*n*	Mean	STD	p*	*n*	Mean	STD	p*	time	group	group × time^a^
GGT [U/l]	R	24	45	56	n.s.	22	23	15	<0.05	22	25	28	n.s.	24	24	30	n.s.	<0.05	<0.01	n.s.
	O	25	33	14		23	36	28		22	33	33		20	23	18				
TG [mg/dl]	R	25	161	72	n.s.	23	125.5	34.6	n.s.	22	112.5	30.8	<0.05	24	103.5	35.2	<0.01	<0.001	n.s.	<0.05
	O	25	125.4	54.4		25	135.7	54.4		22	94.3	18.4		21	74.9	21.5				
Platelets [G/l]	R	25	261	43.6	<0.05	22	279	57	n.s.	22	275	60	n.s.	24	280	60	n.s.	n.s.	<0.001	n.s.
	O	25	294	54		25	266	53		22	270	58		20	274	67				
PT [%]	R	24	113	27	n.s.	22	110	26	n.s.	22	106	29	n.s.	24	113	29	<0.05	n.s.	n.s.	0.05
	O	25	125	16		25	103	20		22	104	19		20	97	17				
hsCRP [mg/dl]	R	24	1.6	2,4	n.s.	23	0.6	0.5	n.s.	22	0.6	1.1	n.s.	24	0.3	0.4	n.s.	<0.001	n.s.	n.s.
	O	25	1.0	0.6		25	0.9	1.3		22	0.4	0.5		20	0.2	0.4				
Glucose [mg/dl]	R	10	7.5	2.6	n.s.	7	5.4	2.1	n.s.	15	3.6	1.4	n.s.	20	3.4	1.3	n.s.	<0.001	n.s.	n.s.
	O	18	3.7	1.4		17	3.9	2.5		14	3.4	3.4		13	2.9	1.9				
LF [%]	R	24	1.1	0.4	<0.001	22	1.1	0.3	n.s.	22	1.1	0.2	n.s.	24	1.0	0.3	n.s.	<0.02	n.s.	n.s.
	O	25	1.2	0.3		25	1.2	0.3		22	1.1	0.3		20	1.2	0.3				
DRR []	R	25	99	16	n.s.	23	94	5	n.s.	22	90	7	n.s.	24	90	8	<0.01	<0.05	n.s.	<0.05
	O	25	99	10		25	94	9		22	86	8		21	86	11				
Insulin [μU/ml]	R	25	26	13	<0.05	23	15	4	<0.01	22	12	6	n.s.	24	10	5	n.s.	n.s.	n.s.	n.s.
	O	25	16	9		25	9	6		22	9	15		21	10	13				
Weight loss [%]	R			–	–	11	15.7	4.3	0.001	19	23.8	6.0	<0.001	23	29.7	9.2	<0.01	<0.001	<0.001	0.001
	O			–		16	23.3	5.5		23	33.5	6.6		16	37.9	6.5				

*R* Roux-en-Y gastric bypass, *O* omega-loop gastric bypass, *GGT* gamma-glutamyltransferase, *TG* triglycerides, *PT* prothrombin time, *hsCRP* high-sensitive C-reactive protein, *LF* liver fat, *DRR* De Ritis ratio, *n.s.* not significant

*T and Mann-Whitney *U* tests comparing RYGB and OLGB at baseline (T0), after 3 (T3), 6 (T6), and 12 (T12) months; **linear mixed model; *time* differences over time course, *group* differences within the groups, *group* × *time* changes over the time course and between group

### Surgical Technique

All procedures were performed by the same surgical team using a laparoscopic approach. Roux-en-Y gastric bypass consists of a longitudinal 30-ml gastric pouch which is anastomosed end-to-side with the jejunal limb and a latero-lateral jejuno-jejunal anastomosis resulting in a biliopancreatic limb of approximately 80 cm and an alimentary limb of approximately 150 cm [5]. Omega-loop gastric bypass is a simplified procedure that consists of a unique gastrojejunal anastomosis between a 30–40-ml sleeve gastric pouch and a jejunal omega loop of 200 cm [5, 19].

### Follow-Up

All patients were followed up before surgery (T0) (on average 2 months), 3 (T3), 6 (T6), and 12 (T12) months postoperatively. Medical treatment was adjusted according to the current needs of the patient. Postoperatively, supplements were prescribed with respect to the available guidelines at that time [3].

### Variables

Following blood parameters were evaluated: total protein, albumin, total bilirubin, alkaline phosphatase (AP), aspartate transaminase (AST), alanine transaminase (ALT), gamma-glutamyltransferase (GGT), triglycerides (TG), prothrombin time, platelets, glucose, insulin, and high-sensitive C-reactive protein (hsCRP) as marker for tissue damage, infection, inflammation, and malignant neoplasia [20]. The NAFLD liver fat score [21], a test to accurately predict non-alcoholic fatty liver disease (NAFLD), liver fat percentage (%) [21], based on fasting glucose, fasting insulin, De Ritis ratio, the presence of metabolic syndrome and type 2 diabetes, and the NAFLD fibrosis score [22], a scoring system consisting of BMI, age, glycemic status, platelet count, albumin level, and De Ritis ratio [22] to separate NAFLD patients with and without advanced fibrosis were calculated. Furthermore, De Ritis ratio, as a marker for severe liver damage [23], as well as homeostasis model assessment of insulin resistance (HOMA2-IR) and quantitative insulin sensitivity check index (QUICKI) [24], as marker for insulin sensitivity, were computed. The presence of metabolic syndrome was defined according to criteria of the International Diabetes Federation [25]. Abnormal liver parameters were defined as an elevation of ALT, and/or AST, and/or GGT levels above the upper limit of normal (ULN) [26]. In order to discuss the clinical relevance of any findings, there was a separate analysis with values >2× ULN and the proportion of results out of the normal range. Age, height, weight, and BMI were determined. Due to the different WL reporting methods in previous studies, we decided to calculate all of the recommended ones [27] to allow easier comparison of the data.

### Statistical Analysis

Statistical calculations were performed by SPSS 20.0 (SPSS Inc, Chicago, IL). Data are shown as means and standard deviations (SD). The hypothesis of variables being normally distributed was tested by Kolmogorov-Smirnov test. Differences in the distributions of variables between Roux-en-Y gastric bypass and omega-loop gastric bypass were tested by a Student’s *t* test, respectively, by Mann-Whitney *U* test for two independent samples. We used repeated-measures ANOVA, using random error (linear mixed model) to assess the effect of time and the interaction for changes in laboratory parameters between the groups, by using different covariance structure models as appropriate and adjusted for baseline values, age, and sex. Moreover, a post hoc analysis with Bonferroni correction was used. Binary logistic regression was used to estimate the odds for type of surgery. Linear regression was used to identify independent variables (e.g., age, gender, preoperative BMI) associated with changes in the evaluated parameters. Spearman’s and Pearson’s correlation coefficients were used to determine associations between changes in relevant parameters. The chi-square test was applied to control whether there are significant differences between the expected and the observed frequencies in categorical liver, cholestatic, and metabolic parameters. Means were compared unadjusted without imputation of missing data. Statistical significance for all analyses was assumed as *p* ≤ 0.05.

## Results

### Liver Function

For AST, a significant group and time difference could be found. ALT dropped in Roux-en-Y gastric bypass, while rising in omega-loop gastric bypass with a significant group and time difference. Moreover, a positive correlation could be found between ALT, surgical method (*r* = 0.406, *p* = 0.006) and De Ritis ratio >1 (*r* = 0.451, *p* = 0.002) at T12. Notably, no correlation between WL, ALT, and AST was observed. These parameters remained in the normal range in both groups (Fig. 1).

Initially, Omega-loop gastric bypass group showed a significant higher count in *platelets*, this difference disappeared at T3. Nevertheless, a difference between the groups (Table 1), as well as a negative correlation with WL (*r* = −0.346, *p* = 0.002) could be detected.

A significant group and time interaction could be found for *prothrombin time* (Table 1) as well as a significant increasing proportion of low prothrombin time in omega-loop gastric bypass at T3 (Roux-en-Y gastric bypass vs. omega-loop gastric bypass; T0: 8 vs. 0 %, n.s.; T3: 5 vs. 8 %, *p* < 0.05). A negative correlation between prothrombin time and WL was found in omega-loop gastric bypass (*r* = −0.543, *p* < 0.001).

No differences appeared in the course of *albumin*, while *protein* diminished significantly over time and group (Fig. 2). A negative correlation between protein and WL in both groups (*r* = −0.428, *p* < 0.001), as well as for albumin and hsCRP (*r* = −0.611, *p* < 0.01) in omega-loop gastric bypass at T6 was found.

### Cholestatic Parameters

In Roux-en-Y gastric bypass, GGT significantly decreased by nearly half of the initial value, while it remained stable in omega-loop gastric bypass and a significant group and time difference could be found (Table 1). Younger patients (<50 years) had a greater chance of lower GGT quartiles over time (odds ratio (OR) 0.52; 95 % confidence interval (CI) = 0.36–0.75; *p* < 0.001; adjusted for initial BMI). A negative correlation between GGT and WL could be seen in Roux-en-Y gastric bypass (*r* = −0.571, *p* < 0.001).

In the whole cohort, a maximum rise in *bilirubin* and AP was found at T3. The values of both remained in the high–normal range until T12 (Fig. 3).

### Calculated Liver Scores

NAFLD *liver fat score* showed a significant higher proportion of NAFLD in Roux-en-Y gastric bypass until T6 (T0: Roux-en-Y gastric bypass 75 % vs. omega-loop gastric bypass 6 %, *p* < 0.001; T3: 57 vs. 8 %, *p* < 0.01; T6: 13 vs. 5 %, n.s.; T12: 0 vs. 13 %, n.s.).

Liver fat percentage showed a similar development with a significant difference over time (Table 1). Over the whole observation period, a strong correlation could be found between liver fat percentage and HOMA2-IR (*r* = 0.616, *p* < 0.001); QUICKI (*r* = −0.623 *p* < 0.001); AST (*r* = 0.506, *p* < 0.001); ALT (*r* = 0.429, *p* < 0.001); and TG (T0: *r* = 0.519, *p* < 0.001) in both groups.

Omega-loop gastric bypass showed a significant higher *De Ritis ratio* at T12 (Table 1) and a significant group and time interaction could be detected. Subsequently, at T12, 54 % in Roux-en-Y gastric bypass vs. 90 % in the omega-loop gastric bypass (*p* < 0.01) had a De Ritis ratio ≥1. Over the observation period, no differences were found in the distribution of individuals with, without, and undefined status of liver fibrosis calculated by *NAFLD fibrosis score*.

### Metabolic Markers

*Glucose* significantly decreased in both groups until T12. *Insulin* was significantly higher in Roux-en-Y gastric bypass at T0, although no group and time interaction could be found. We observed a significant group difference for HOMA2-IR from T0 to T6 and a significant group and time effect (Fig. 4), whereby values were higher in Roux-en-Y gastric bypass. QUICKI was significantly higher in omega-loop gastric bypass patients at T3, T6, and T12; however, no group and time interaction could be detected (Fig. 4).

TG showed a significant reduction over time in both groups. Worth mentioning is a significant initial increase of TG in omega-loop gastric bypass, which finally ended up in a greater reduction. For all above-mentioned metabolic markers, a negative, respectively, for QUICKI positive, correlation with WL in both groups could be seen (glucose: *r* = −0.451, *p* < 0.001; insulin: *r* = −0.379, *p* < 0.001; HOMA2-IR: *r* = −0.520, *p* < 0.001; QUICKI: *r* = 0.540, *p* < 0.001; TG: *r* = −0.418, *p* < 0.001).

hsCRP showed a reduction over time with lowest levels in both groups at T12. Notable correlations were found with WL (*r* = −0.293, *p* < 0.001) over time in both groups, respectively. AST (*r* = 0.527, *p* < 0.001); AST >2× ULN (*r* = 0.501, *p* = 0.001); ALT (*r* = 0.358, *p* = 0.02); ALT >2× ULN (*r* = 0.488, *p* < 0.01); and glucose (*r* = 0.474, *p* < 0.01), in both groups at T0.

### Weight Loss

WL (mean ± SD) was 30 ± 9 vs. 38 ± 7 % (%BMI loss), which equals a percentage excess WL (%EWL) of 94 ± 36 vs. 127 ± 31 % or percentage excess BMI loss (%EBMIL) of 67 ± 22 vs. 88 ± 16 % in Roux-en-Y gastric bypass vs. omega-loop gastric bypass after 12 months. Using the binary logistic regression, an increased chance for greater WL in omega-loop gastric bypass was shown (OR = 1.19; 95 % CI = 1.11–1.27; *p* < 0.001).

## Discussion

The superiority of omega-loop gastric bypass over Roux-en-Y gastric bypass in terms of WL was demonstrated in previous studies [5, 6], as well as in our study. We did not observe any influence of gender, age, or preoperative BMI on WL regardless of the procedure. Studies reported an EWL percentage range from 59 to 63 % in Roux-en-Y gastric bypass [5–7, 19, 28–30] and 65 to 89 % in omega-loop gastric bypass [5, 6, 30]. Compared to this data, we observed a larger WL in both groups. The difference might be explained by differences in the length of the bypassed small bowel with a differing malabsorptive effect. In addition to the superior WL in omega-loop gastric bypass, we might assume additional beneficial effects, as elevated transaminases, cholestatic and metabolic parameters improve driven by WL [11, 31, 32].

WL is currently an effective treatment of NAFLD and non-alcoholic steatohepatitis (NASH) in patients with obesity [11, 32, 33]. Several studies investigating the effect of bariatric surgery on liver enzymes and NAFLD have shown an improvement of serum transaminases and hepatic histologic features after surgery [8–11]. However, so far, no current data is available for omega-loop gastric bypass and liver status. Certainly from previous literature, it became evident that rapid WL after bariatric surgery can also adversely affect the liver [11]. This could be attributed to the already pre-existing degree of fibrosis which subsequently worsened by rapid WL due to the bariatric procedure and/or due to a lack of adequate supplementation of macro- and micronutrients [11, 34].

In our study, the increase of liver transaminases (ALT, AST) was greater in omega-loop gastric bypass than in Roux-en-Y gastric bypass with a maximum rise after 3 months. These findings are comparable with two studies of Wolf AM et al. [9, 35]. The values of AST and ALT successively decreased and finally fell below those of T0 in Roux-en-Y gastric bypass, whereas both parameters remained above the preoperative values in omega-loop gastric bypass. The results remained within the normal range in both groups and during the whole observation period. Nevertheless, even slightly increased but still normal aminotransferase concentrations are related to an increased risk of death from liver disease [36]. Significant liver disease (i.e., bridging fibrosis and cirrhosis) may be accompanied by mild transaminase elevations [37], as seen in our data. GGT, as well as AST, are associated with higher risk of cardiovascular disease (CVD), particularly in young individuals [38, 39]. GGT is known as a marker for sub-clinical inflammation, oxidative stress, visceral, respectively, intrahepatic fat and therefore for insulin resistance [38]. Our data shows a rapid reduction of GGT in Roux-en-Y gastric bypass and a temporally delayed one in omega-loop gastric bypass, especially in younger patients, who might benefit most from a reduction [39]. Bilirubin and AP were both increasing after surgery and remained above the baseline level after 12 months. Moreover, a dependence of bilirubin and AP with WL could only be seen in Roux-en-Y gastric bypass. These findings are contrary to previous data [40]. Furthermore, we found a clear positive association between WL and the improvement of parameters for insulin resistance, insulin sensitivity and liver fat percentage in both groups. Interestingly, in comparison to other studies [8, 32], we could not find a correlation between AST, ALT, and WL.

Noteworthy, the proportion of patients with higher De Ritis ratio was significantly greater in omega-loop gastric bypass after 12 months. Furthermore, omega-loop gastric bypass presented a significantly higher level of ALT, lower level of protein with a negative correlation with WL, prothrombin time, and platelets, which are also indicators for liver function [41], at T12. The transient elevation of liver parameters in patients after bariatric surgery [11, 34, 35] and during a low-calorie diet without bariatric surgery [31] were previously described. Different hypotheses explaining these changes in hepatic and metabolic parameters exist, whereas it is most likely that the triggers are multifactorial. Mild and transient changes in liver histology, which themselves could be induced by the modifications in liver physiology such as the increased ketone production or active mobilization of lipids from adipose tissue and hepatocytes during calorie restriction resulting in potentially toxic intermediate metabolites such as free fatty acids and ceramides, could be a key mechanism [31, 42]. Furthermore, rapid mobilization of intra- and extrahepatic fat stores are probably associated with deficient protein intake during WL and may contribute to aggravation of pre-existing liver disease [34]. Moreover, the trauma of surgery associated with an increase in production of stress hormones, oxidative stress, and inflammatory responses is associated with reduced hepatic function [9]. Importantly, we could not detect a correlation between liver transaminases and WL.

We found higher levels of several metabolic parameters (insulin, HOMA2-IR, liver fat percentage) at T0 in Roux-en-Y gastric bypass despite of matching our groups for age, sex, and BMI. Those differences disappeared at T12, and all of them were positively correlated with WL. Consequently, both surgical groups showed a positive metabolic outcome due to WL.

A limitation of the current report is the small sample size, due to the restricted availability of non-diabetic omega-loop gastric bypass data. Nevertheless, this sample is well characterized and the laboratory data is almost complete, which strengthens our study. The data represents a novelty, because up to now, to our current knowledge, no study has assessed the differences in liver, cholestatic, and metabolic parameters in Roux-en-Y gastric bypass vs. omega-loop gastric bypass. Another limitation is the lack of liver biopsies, which might give us better insight into the liver situation preoperatively and during the postoperative period. Therefore, we cannot exclude the presence of relevant liver injury at baseline, which possibly could explain the results in omega-loop gastric bypass at T12.

In conclusion, omega-loop gastric bypass results in greater WL and deterioration of liver parameters in the first year after surgery with no association to WL. It remains unclear what induces the bimodal alteration in liver transaminases and cholestatic parameters during the early postoperative phase and also after 12 months. Given the potential for worsening fibrosis following bariatric surgery, patients should be monitored closely and continue to undergo through hepatological workup, including non-invasive testing for fibrosis or, even liver biopsy. More research in this field, including histopathological data, is needed to define clinical relevance of these findings on hepatic outcome in terms of deterioration of non-alcoholic steatohepatitis and fibrosis and to characterize patients at risk. In both groups, improved outcome in terms of insulin resistance and sensitivity could be found in correlation to higher WL and lower liver fat percentage, respectively.
